# A Performance Management System for Long-Term Germplasm Conservation in CGIAR Genebanks: Aiming for Quality, Efficiency and Improvement

**DOI:** 10.3390/plants10122627

**Published:** 2021-11-29

**Authors:** Charlotte Lusty, Janny van Beem, Fiona R. Hay

**Affiliations:** 1 Global Crop Diversity Trust, Platz der Vereinten Nationen 7, 53113 Bonn, Germany; janny.vanbeem@croptrust.org; 2 Department of Agroecology, Aarhus University, Forsøgsvej 1, 4200 Slagelse, Denmark; fiona.hay@agro.au.dk

**Keywords:** seed quality management, long-term conservation, quality management system (QMS), performance management, genebanks, standards

## Abstract

UN Sustainable Development Goal 2 Target 2.5 focuses on the conservation of genetic diversity in soundly managed genebanks. In examining the term “soundly managed”, it becomes quickly evident that there is much more to long-term conservation than placing samples of seeds or other germplasm in long-term conservation conditions. There are several important factors that determine whether germplasm samples will remain viable in storage for long periods of time. To manage these factors efficiently and effectively, genebanks require sound data and quality management systems. The CGIAR Genebank Platform, coordinated by the Crop Trust, put in place a number of mechanisms that enabled effective online reporting, performance management, quality management, audit and external review and validation. These mechanisms do not conform to the usual monitoring systems put in place for research programs and have only been possible thanks to the flexibility of CGIAR in recognising that the genebanks were exceptional. As a result, in the past 10 years, CGIAR genebanks have significantly improved their performance and the conservation status of collections.

## 1. CGIAR Genebanks, Germplasm Conservation and Genebank Standards

The CGIAR genebanks safeguard some of the largest and most widely used collections of crop diversity in the world, critical to attaining the UN’s Sustainable Development Goals (SDG) to end hunger and improve food and nutrition security [1,2]. Most CGIAR genebanks are strategically located in centres of crop diversity, resulting in the collections being founded on a broad and rich representation of diversity from the primary crop genepools and, in some cases, a continued exchange with traditional communities cultivating landraces or even domesticating semi-wild materials. The collections that CGIAR manages have grown over five decades or more in a relatively organic fashion with the gradual introduction and occasional peaks of expansion resulting from collecting missions, donations from partners and materials generated by breeders and researchers. As a result, the collections represent a worldwide diversity of landraces, heritage varieties, crop wild relatives, improved varieties and, to a lesser extent, breeding or research materials for specific mandate crops (Table 1).

The collections and their contents may be reviewed online through the global portal, Genesys (www.genesys-pgr.org last accessed 24 November 2021). The importance of the collections is recognized in international policy through agreements (Article 15) signed in 2006 between each CGIAR Centre and the Governing Body of the International Treaty on Plant Genetic Resources for Food and Agriculture (Plant Treaty) [4]. Centres are obliged to make collections and associated data under their management available under the Multilateral System (MLS) of Access and Benefit-sharing of the Plant Treaty. CGIAR genebanks act as a major source of international germplasm exchange and, together with CGIAR breeding programs, were responsible for close to 90% of the reported distributions under the Plant Treaty [5]. Between 2012 and 2019, the CGIAR genebanks distributed more than 850,000 samples of germplasm to 163 countries in response to requests [3]. Only the US Department of Agriculture National Plant Germplasm System distributes more germplasm, about 250,000 samples yearly, of which about a quarter is distributed internationally.

The UN’s 17 SDGs were agreed by world leaders in 2015. SDG 2 has eight targets with the aim to end hunger in the world. Target 2.5 specifically calls on countries and institutions to maintain genetic diversity in food production, including in “soundly managed” seed and plant banks [6].

Over many decades, hundreds of institutes, universities, research groups and communities have carried out research, breeding and collecting and, as a result, have invested in storing seeds in freezers or cold rooms. Which of these multitudes of efforts constitute a formal seed or plant bank (‘genebank’) is not easily determined. Most countries have formally designated a national genebank under the management of national agricultural research organizations, but there are many other formal and informal collections and gardens, large and small, managed within the public or private sector. Seeds in storage ultimately perish if not planted out and regenerated before viability is lost. The genetic composition and the rare alleles present in a seed sample will be increasingly lost over time as more and more seeds in the sample lose viability. Germplasm conservation in genebanks aims to maintain the viability and genetic integrity of collected samples in storage for the longest time that is biologically possible. Multiple guidelines have been published over the years to share best genebank practices [7,8,9]. Germplasm processing for conservation involves several critical steps that can have a prolonged and cumulative influence on the viability and quality of the seeds in storage and their use in the future, as well as on the efficiency of future operations [10]. The critical point, therefore, is not defining what is a seed or plant genebank but in determining what is “soundly managed”.

Genebanks serve different purposes and clients. There are many different options for localised communities to save seeds for sharing and planting in the near term. For prolonged conservation and for medium to large collections (e.g., more than 10,000 accessions), the requirements in terms of capacity and processes are considerably more extensive. Any institution wishing to manage a genebank today is ably guided by published Genebank Standards, as endorsed by the FAO Commission on Genetic Resources for Food and Agriculture (CGRFA) [4,11], which provide quantitative thresholds, principles, and critical points for genebank management. There is no formal system of monitoring or measuring compliance with the FAO Genebank Standards, but institutes managing collections have an option to undergo certification or accreditation with the International Standards Organization (www.iso.org; last accessed 24 November 2021) or the International Seed Testing Association (for specific tests; www.seedtest.org; last accessed 24 November 2021). A small number of genebanks have pursued a quality management system (QMS) certification through ISO 9001:2015 which applies to businesses or organizations that seek to ensure that their products or services meet customer needs. While there are benefits to the certification, the Genebank QMS described below includes additional aspects that are outside the scope of certification, e.g., adherence to the International Treaty on Plant Genetic Resources for Food and Agriculture (ITPGRFA) (https://www.fao.org/plant-treaty/en/; last accessed 24 November 2021), the International Plant Protection Convention (www.ippc.int; last accessed 24 November 2021) and the Convention on Biological Diversity and its Nagoya Protocol (www.cbd.int/; last accessed 24 November 2021).

So, what evidence is there of sound management or even that reported accessions really exist? Requesting and receiving seeds from genebanks is one way of telling whether they exist and are viable—but relying on feedback from users is leaving it too late and is hardly feasible for very large collections. If taxpayers are to fund and depend on key institutions, such as CGIAR and other international or national genebanks, to safeguard agricultural diversity for future generations, then the large body of stakeholders (including donors, depositors and users) need to have some confidence that the methods in use meet appropriate standards and will achieve long-term conservation objectives. Furthermore, we need to know that institutions are actively improving processes and incorporating appropriate technologies and approaches to conserve germplasm efficiently. Engels and Ebert [12] provide an exhaustive critique of current conservation methods currently deployed in genebanks with many points that should be addressed.

By describing our experience with CGIAR genebanks, we endorse the view that a comprehensive system of quality and performance management is necessary to provide assurance that key genebanks are complying with FAO genebank and other relevant standards and to provide a continuous mechanism for exchanges and improvement. We describe the challenges that lie behind long-term conservation objectives and the need for serious investments to meet such a commitment, perhaps more than many actors anticipated at the time of collecting seeds and putting them in storage. Such long-term conservation objectives can only be managed through long-term and consistent quality management [10]. In our experience, a quality and performance management approach has helped to manage backlogs and to avoid mistakes, losses and duplication of effort, as well as facilitating continuous improvement. The requirements of long-term conservation objectives should be fully understood by all institutions that aim to accommodate such long-term commitments within a predominantly research-oriented program of work.

## 2. Why Long-Term Conservation Is Not as Easy as One Might Think

The effective conservation of seed germplasm samples with the aim of maintaining genetic integrity involves the complex interaction of various factors, including biological characteristics intrinsic to the species, the quality of the seeds and the conditions of the seeds’ storage [10,11,13]. If seeds are stored in cold storage without attention to drying and testing processes, packaging, monitoring and maintenance of conditions, then only with luck will they maintain their viability to be germinated some years later. If the genetic diversity of a collection is to be conserved over many decades and some of the unique and rare traits occurring in the gene pool are to be retained for future research and breeding, then an intensively evidence-based approach to long-term conservation is required. Four main factors explain why long-term conservation efforts specifically for seed collections require considerable investment:

(1) **Biological factors:** Although seed storage has been a fundamental aspect of agricultural practice for millennia, the long-term storage of dry seeds at low temperatures has only been applied scientifically for relatively few decades. Seeds of some species are predicted to remain viable for perhaps hundreds of years under long-term conditions in a genebank (typically at 3–7% moisture content and −20 °C temperature) [13]. Further, many genebanks have now reported the long-term maintenance of high levels of viability, for many (but not all) seed lots (samples) of at least some crop and forage species, over the course of the genebank’s existence [10,14,15]. However, there are examples where seed lots have lost viability relatively quickly or abruptly during genebank storage [10,16,17]. As more viability monitoring data becomes available, seeds of more species, particularly those of crop wild relatives, forage and tree species, may be found to be relatively short-lived in conventional genebank storage. In conclusion, the majority of crop species are thought to exhibit orthodox storage behaviour, tolerating drying to low moisture contents and remaining viable in storage for extended periods, but until more evidence is gathered through viability monitoring of actual seeds in storage, we do not know the extent to which certain groups of genotypes or species are not orthodox. Cryopreservation may be more suitable for ‘minimally orthodox’ seeds with very short lifespans in conventional genebank storage, though it may also be possible to improve their storage potential to some extent. Cryopreservation itself, however, is an expensive commitment.
(2) **Seed quality management:** Seed longevity is a highly plastic trait, affected by factors such as the environment during seed production, the timing of seed harvest and how the seeds are processed after harvest [18,19,20]; maximizing seed storage potential requires experience, experimental approaches to optimizing protocols and attention to detail. Drying seeds appropriately before storage, maintaining conditions throughout storage and careful practice when repeatedly accessing stored seed lots for distribution or viability monitoring have a large and direct impact on the number of years that seeds remain viable in storage. Different types of collections pose very different challenges. Working with a small, diverse collection that is frequently being accessed poses different challenges to working with a very large single crop collection where efficiencies of scale mean seed lots can be processed at higher throughput, making the whole genebank operation more efficient.
(3) **Sustained data management needs:** Maintaining a seed collection for both active use and conservation demands a major investment in documentation if these two different roles are to be fulfilled efficiently and effectively. All samples should be labelled to ensure that the right seeds are in the right place at the right time. Over long periods of time and many interventions, mislabelling events are inevitable. A study of lettuce accessions conserved at the Centre for Genetic Resources the Netherlands (CGN) found the highest rates of non-authenticity for samples that date back to the earliest collections but even those more recently conserved materials (post-1960) showed around 10% mislabelling [21]. Barcoding is believed to eliminate some mislabelling errors thanks to the automated production of identification labels that reduces the human error caused by writing labels by hand. The value of barcoding, however, is extremely limited without being fully integrated into a comprehensive seed inventory data management system that follows the complete genebank workflow from the introduction of a new acquisition to the point of its storage, including activities that may be undertaken by associated teams or laboratories, such as health testing and fieldwork. Data on post-harvest treatments, initial viability and the detailed procedures followed at the time of germplasm processing and storage are basic pieces of information required to manage a seed lot in the long-term. The data management system should not only store these data points, or references to them, but should follow the operational workflow providing prompts if data are not entered correctly and assisting quality controls that underpin the smooth running of the genebank [22]. In the long term, staff will change and an effective data management system is fundamental to enabling collection management to pass safely from one pair of hands to another. In the future, such management systems could evolve with smarter tools and algorithms to take some of the burden of data entry and decision-making away from staff, but for now, even the basic functionality is an ambition for most genebanks.
(4) **Sustained infrastructure needs:** Finally, conserving with a long-term perspective means that a one-off investment in a cold room is not enough. Cold rooms may have a service life of up to 50–60 years but other critical equipment such as door seals, cooling systems, incubators, driers, and temperature controls have a shorter service life and require maintenance, replacement and backup on a regular basis.

These four factors apply to all genebanks, but the long-term conservation of ‘non-seed’ collections is yet more challenging. A relatively recent assessment of vegetatively-propagated crops in ex situ conservation estimated that there are at least 400,000 accessions in genebanks worldwide, of which 75% are held in the field and 15% in tissue culture collections [23].

Field collections are notoriously difficult to maintain over many decades because actively growing accessions need repeated monitoring and intervention throughout the year and, in many cases, yearly planting and harvesting to ensure the continued health of the plants and to manage the effects of weather events and pests and diseases [9]. There is little published information on how long accessions have been successfully maintained in field collections for conservation purposes. There are, however, numerous cases of field collections that have been destroyed due to typhoons, drought, disease, change in land management, loss of interest in “low-income” crops that have been reported to the Global Crop Diversity Trust (Crop Trust). There are, also, a few examples of field collections that have stood the test of time, including stands of cacao and coffee in Turrialba, Costa Rica, maintained by the Tropical Agricultural Research and Higher Education Center (CATIE), which date back to 1947. Many botanic gardens will also include collections and old specimens of crop species, but normally in very low numbers. All such collections remain vulnerable and prone to losses.

Tissue conservation in vitro requires equally, if not more, intensive management and infrastructure. This approach is frequently used more for the propagation of planting materials than for conservation objectives. Although there are significant numbers of accessions reported to be in tissue culture, it is not known what proportion of these is conserved using slow-growth conditions for conservation purposes.

Neither field nor in vitro conservation approaches are sufficiently reliable or optimised to be considered a “long-term” conservation approach [24]. Considerable investment, however, has been made in cryopreservation [25], a technology that is coming of age for certain crops (e.g., potato, garlic, banana, apple) but not yet as a standard technique for all vegetatively-propagated crops and non-orthodox seed crops due to a general lack of capacity both to develop and optimize the specific protocols required for more challenging species, and sometimes genotypes within species, and to implement cryopreservation on a large-scale for whole collections. In 2017, just 17 genebanks were identified as actually using cryopreservation as a conservation procedure [23]. The costs of implementation and staff training remain insurmountable hurdles for most genebanks wishing to take up cryopreservation. A globally coordinated initiative to build capacity would help to secure such collections on a long-term basis [23].

## 3. Managing Genebanks for Long-Term Conservation Objectives

Even where sound processes and data management are established and conditions for long-term conservation are optimized, it is very common for genebanks to be inadequately resourced to carry out the required processes on all the germplasm in the collection on a continuous basis [10]. Taking account of general guidance on viability monitoring, seeds in long-term conservation should be re-tested for viability every ten years or more frequently if short-lived—that is unless there is evidence that the viability of accessions will remain above the accepted threshold for more than 30 years in which case longer periods between re-testing may be appropriate [11]. Seeds in medium-term storage will potentially need more frequent re-testing. In any case, given such standards, an average genebank managing 30–50,000 (the average number of accessions in 71 surveyed national genebanks worldwide was 41,342 in 2014 [26]) accessions (and perhaps twice as many seed lots) will need to conduct viability tests on thousands of seed lots per year (i.e., at least 3–5000 seed lots or 10% of the collection in long-term storage). Some seed lots will fall below the accepted threshold of viability, triggering a demand for regeneration. An FAO survey of 488 national and international genebanks in 2014 revealed that 5.7% of the collections were regenerated that year and an additional 137,000 accessions needed to be regenerated but were unable to be planted out because of insufficient funds to carry out the work [26]. Likewise, a review of 26 crop conservation strategies identified regeneration backlogs as a critical issue for all types of crop germplasm in all regions. For one of the easiest crops to conserve, wheat, lack of regeneration was described as probably the single greatest threat to the safety of key collections [27]. Lack of funds and trained staff is identified as a perennial constraint. The situation worsens with every passing year, as the number of accessions that require viability re-testing, regenerating and other basic operations increases. The words of Lewis Carroll’s Red Queen, “*it takes all the running you can do, to keep in the same place*” could have been written for genebanks.

In 2012, all the CGIAR genebanks, with one exception, had backlogs of materials that required regeneration or processing and were essentially unavailable for distribution unless seeds were tested or regenerated. CGIAR conserved 708,761 accessions in 2012, but only 66% were physically available and 55% safety duplicated in two locations, including the Svalbard Global Seed Vault [28].

The Crop Trust, as coordinator of the CGIAR Genebanks Research Program (2012–2016), put in place a monitoring system for operations across all CGIAR genebanks. The system comprises five elements: (1) performance targets, (2) online reporting, (3) a genebank quality management system (QMS), (4) system-level SOP documentation audit, and (5) external review and validation.

### 3.1. Performance Targets

Targets related to general operations and performance provide a measurable goal and help display the distance required to get there. There is much discussion on the application of performance targets in private and public sectors, and the gaming or perverse incentives that they provoke, especially if linked to financial rewards. The genebank performance targets (Table 2) follow S.M.A.R.T. principles (specific, measurable, achievable, relevant and time-bound) and are designed to measure the level of backlogs and, therefore, the level of activity and time required for individual genebanks to reach a steady-state of operation where backlogs are reduced to less than 10% of the size of the collection. The Passport Data Completeness Index (PDCI) is used as a key performance indicator to measure the level of completeness of passport data associated with genebank accessions [29]. The PDCI range is between zero and ten and is periodically monitored to assess improvements in data quality over time.

Additional data are also collected on genebank activities and germplasm distribution, but none of these indicators are used as targets for various reasons including consideration of the kind of incentive that would be created. Activities to optimize collections were planned and funded, and annual workplans and reports were designed to monitor the progress in improving the status of the collections.

As a result, in 2020, CGIAR reported the availability of an additional 134,994 accessions or 82% of the total collection [30]. This progress occurred in a context whereby, on average, 100,000 samples were being distributed annually from the genebanks and one of the largest collections, at ICARDA (150,000 accessions), had to be regenerated from safety duplicated samples deposited in the Svalbard Global Seed Vault after the genebank was forced to move from Syria in 2012. There is no doubt that the targets focused efforts and resources towards addressing regeneration and processing backlogs.

### 3.2. Online Reporting Tool (ORT)

Underpinning the performance targets is a comprehensive monitoring system with some 200 data points related to the status of the collections, germplasm distribution, other genebank services, activities of the genebank with relation to quality and risk management, cryobanking, capacity building events and activities to respond to review recommendations. The data are received from each genebank, reviewed by the Crop Trust as coordinator of the responsible CGIAR program, and subsequently reported to CGIAR and Crop Trust donors and management, as well as the Governing Body of the Plant Treaty and the CGRFA.

Online reporting is hardly a novelty. Many organisations have moved towards digital systems to manage workplace processes, including project management and annual reporting. There are, however, several points to make about the process by which annual reports were submitted by CGIAR genebanks online that contributed to the improved quality of data and a stronger incentive to address performance targets. Firstly, the data fields requested in the online reporting tool (ORT) were not easily answered through existing genebank database systems, which led to considerable time being demanded by genebank teams to manually calculate figures for each submission. For instance, requesting “Total number of accessions with acceptable viability” or “Total number of accessions with acceptable seed number” may require downloading results for seed lots from multiple sources and years and a reconciliation at accession level. This highlighted the lack of a coherent management tool to assess inventory data at the collection level and to respond to such questions with the click of a button. While no such capacity existed, the bespoke ORT software has two specific features that play a significant role in improving the quality of data submitted: (1) online correspondence between the submitter and the reviewer is linked to each individual question, which allows real-time clarifications and revisions to take place and be stored; and (2) a data quality control process is imposed on individual questions so that the report submission is blocked until specific answers are revised. The reporting process is intensive but contributes substantially to data quality regarding the collection status and to a greater understanding of the performance targets and indicators across genebank teams. In addition, trend analysis of ORT responses was used to monitor indicators and other metrics to evaluate the genebank’s performance over time. As such, the tool relied on historical analysis to plot trajectories and make funding decisions or take corrective and preventive actions when necessary. This type of dynamic reporting tool underpins the understanding of and adherence to the performance targets and monitoring system.

### 3.3. Genebank Quality Management System (QMS)

In 2014, the CGIAR Genebanks program adopted a QMS approach as a means to formally implement and communicate the standards by which CGIAR genebanks operate. Known as the “Genebank QMS”, the approach develops a unique resource of documents, policies and scientific practices that comply with regulatory policy, genebank standards and other relevant standards. The Genebank QMS provides the framework for the management of the genebanks and the way that they are monitored, audited and externally reviewed. It offers distinct advantages over other forms of quality management by being:

- Based on specific genebank standards and performance targets rather than generic standards.
- Holistic and implemented along the entire genebank operation, from acquisition to distribution, rather than for selected procedures or processes.
- Internally driven with collective and individual goals that lead to better technical performance within the individual genebank’s operation.
- Efficient in terms of the amount of paperwork required following implementation and establishment of the QMS.
- Dynamic and allowing the integration of topical issues affecting genebank management, including protocol optimization, policy changes and emerging risks.
- Homegrown and easily tailored and rendered practicable to the unique situation of each genebank.
- Adaptable to a network of genebanks with templates and elements that can be shared and harmonized where appropriate across countries, crops and conservation systems.

**Figure 1:**
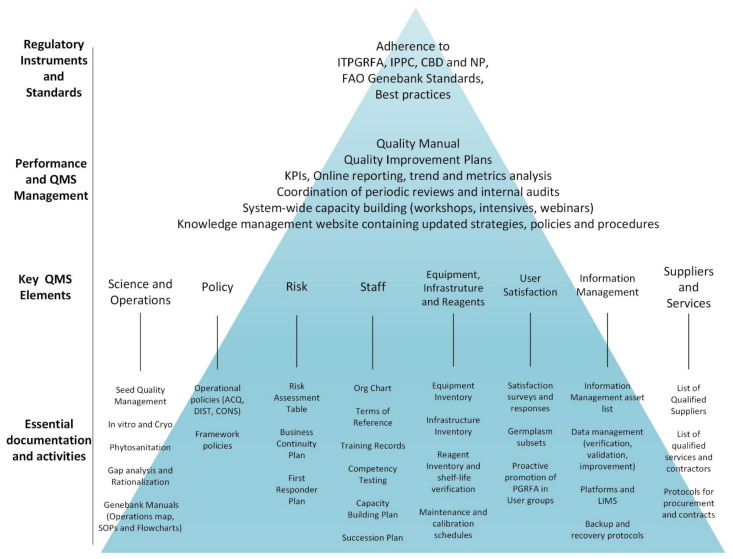
Key elements and regulatory framework of the Genebank QMS.

At the core of the Genebank QMS are eight coordinated elements that serve as building blocks for quality management (Figure 1). Emphasis is placed on the science of conservation to provide an understanding of the underlying principles for adopted processes, including factors affecting seed longevity, cost-efficient ways to conserve genetic resources and use of the collections. The science and the conservation processes are mutually reinforcing, and researchers and curators work together to optimize processes and drive each other forward in the improvement cycle.

Standard operating procedures (SOPs) enable genebanks to comprehensively document, validate, share and improve their operating procedures. The genebanks, therefore, have the possibility to develop and adopt shared policies and approaches for certain processes (e.g., acquisition, distribution, processes for the same crops, etc.) across the system. CGIAR may also put in place certain technical and operational standards and principles that go beyond the published FAO standards, (e.g., CGIAR safety duplicates seed accessions in two locations, uses barcoding for labelling, follows specific standards for cryobanking to name a few examples).

Each individual Centre has the primary responsibility for developing and implementing its own SOPs and QMS, while at the system level the templates, targets, training and auditing are common across genebanks. Agreement to support and commit time to QMS must be attained from the highest level of Center management and from departments throughout the organization, from human resources to procurement. This is essential to ensure that the relevant staff are able to implement core QMS practices (e.g., calibrating equipment, implementing access controls, ensuring health and safety measures).

Staff have the responsibility of reviewing and updating genebank procedures and are encouraged to use their expertise and knowledge to suggest methods for improvement in their work areas. Centers may appoint a quality manager to coordinate QMS activities and report to upper management about QM progress. The tasks carried out by the quality manager may include overseeing the implementation of QM standards and processes by all staff, managing the audit program, reviewing user satisfaction surveys, identifying critical quality control points and preventive measures, monitoring procedure verification, monitoring equipment inventories, and prioritizing risk management and staff training.

As the QMS prompts staff to document the steps actually implemented, i.e., going beyond the function of generic guidelines and giving details of what happens if expected outcomes do not occur, the SOPs can be highly individual and can vary substantially in detail from one SOP to another and from one genebank to another. As the QMS matures and staff see its worth, so the SOPs become more sophisticated with links to additional policies, instructions, and other appendices. It can be challenging to balance the desire to document every detail against the need to have a document that acts as an easy-to-understand reference source or guide.

In addition, fostering collaborations with genebanks outside the CGIAR is widely considered a key strategic goal. The implementation of a QMS and the development of SOPs and other key documents has provided an opportunity for long-serving staff to share their SOPs and expertise with genebanks with little or no documented procedures. In addition, SOPs can be effective teaching tools to disseminate information on best practices and international policies to partners and those genebanks requiring capacity development. Where genebanks manage the same or similar crops, the respective SOPs may be compared and aligned as a means to improve efficiency or level up processes across locations. This can also occur with genebanks outside CGIAR, who have decided to adopt the genebank QMS (e.g., the World Vegetable Center).

### 3.4. System-Level SOPs Documentation Audit

Documentation audits are executed on documented information only and are undertaken by independent consultants with quality management and relevant technical expertise. They are the first step in assuring that individual genebank SOPs are properly documented and adhere to relevant treaties, standards and agreed principles. In the Genebank QMS, SOPs, including decision trees, monitoring schedules and related policies, are checked for compliance with the FAO Genebank Standards, the terms and conditions of the standard material transfer agreement, international standards for phytosanitary measures, international rules for seed testing and other relevant best practices that are agreed within the CGIAR genebank community. The system is relatively agile, with recommendations expected to result in improvement within two months of the initial audit and clearance conferred within four months. The resulting SOPs are also subject to language editing and translation for improved clarity. In addition, the SOP template is designed so that the core of the procedures may be extracted to be part of a genebank manual for publication and general circulation.

### 3.5. External Review and Validation

The first phase of CGIAR genebanks external review began in 2012 and followed a relatively generic approach, whereby two or three experts were asked to make physical visits to individual genebanks and undertake a review based on a small number of relatively broad objectives. The second phase of external review, which started in 2018, however, was able to profit from a vast amount of new information provided in the six years of detailed annual reporting by the genebanks against performance targets and the documentation and auditing of the SOPs. The reviews followed a standard format and involved an extensive document review before the site visit, including review and discussion of the key SOPs, annual report submissions, previous review documents, as well as a self-assessment by the genebank staff.

The review performs an important audit function by validating the actual implementation of specific SOPs by genebank staff on the ground through demonstrations or other evidence. One step in the review process involves checking the inventory data in the live database and the physical samples in the cold rooms of a number of randomly-picked accessions. The format of the reviews allows their implementation even remotely during the pandemic.

Thanks to the QMS and annual reports, the second phase of the review was significantly more evidence-based and in-depth than the first. Weaknesses in data and processes that were not at all evident in the first phase of the review, were easily identified in the second. By contrast, the reviews provided fewer strategic or subjective recommendations on the general direction in which the genebanks were going. The recommendations were incorporated into recommendation action plans (RAP) that feed into the individual genebank’s improvement and QMS development.

## 4. Concluding Remarks

In summary, long-term conservation is a more challenging objective than may be anticipated by many institutions that have stored germplasm in cold rooms. There are several critical requirements of any genebank conserving genetic resources specifically for the long term. Genebank processes involve sustained activities that are basic requirements for long-term conservation, such as viability monitoring and regeneration for seed collections and cryopreservation for vegetatively propagated crops and detailed data management for both. A large number of under-resourced genebanks require more investment to train staff, improve equipment and facilities, support operations and improve data management systems. Key to such investments is the commitment to performance and quality management.

The Genebank QMS does not provide a formal quality certification system, but through the adoption of standard templates and approaches across the group of genebanks and its integration into system-level auditing and review, it assists institutes in developing a recognizable QMS that can be tailored to suit a variety of crops, locations, conservation methods and budgets. Effectively implemented and monitored, the QMS leads to improved administrative, technical and operational performance and the assurance that international standards are being met [31]. Genebank users, regulatory bodies and donors may depend more confidently on the Genebank QMS to recognize and confirm the competence of the genebanks. 

After the experience with CGIAR genebanks, we are persuaded that institutes that manage globally important collections for long-term conservation objectives require:

- Either active conservation research projects or association with a university to address research questions concerning recalcitrant or difficult-to-conserve species, improvement of procedures and introduction of new technologies.
- A Genebank QMS with detailed SOPs and system by which they are regularly updated, reviewed, audited and validated.
- A data management system that not only facilitates inventory management but also supports the genebank workflow and quality control and the overall monitoring of the collection.
- An overall monitoring, reporting and review system that incorporates the QMS approach and provides appropriate incentives for genebanks to strive for improvement and efficiency, and to reach and maintain performance targets.
- Adequate facilities and equipment that are accessed only by selected personnel, regularly calibrated and maintained, backed up and replaced prior to reaching the recommended service life.

It is worth noting that such requirements are not easily accommodated within typical research projects or programs. While this may seem an obvious point to make, the fact remains that most genebanks are part of research institutions and are inevitably subject to monitoring and funding systems that are designed to manage research projects. Genebanks are, therefore, obliged to develop budgets and proposals based on research questions and outputs, theories of change and short-term impact for beneficiaries. Within a strongly research-oriented environment, there will be little incentive for genebanks to invest in quality management processes, to share procedures with “competitors” or to seek efficiencies, let alone work towards long-term conservation goals. Since 2012, CGIAR has recognised that the genebank program is unique and requires a bespoke reporting system, which has been further endorsed by two reviews. [32,33]. This support has allowed a unique genebank performance and quality management system to develop and flourish, which we believe has strongly reinforced the long-term conservation objectives of the genebanks and their resilience for the future.

Indeed, CGIAR genebanks have been able to reliably distribute germplasm over the past five decades thanks to their long-term commitment to conserving these collections. They benefit from a high level of operation, modern facilities, field sites, equipment, backup equipment, trained staff and they have had relatively secure funding, at least over the past 10 years. All of which feeds into CGIAR genebanks being secure places for long-term conservation and in some cases the last refuge for landraces and species that have now disappeared from farmers’ fields or in situ locations. Indeed, the importance of having multiple layers of good practice and policy, including large-scale safety duplication, production of good quality seeds, sound operating procedures, strong partnership with national agricultural research systems and trained and dedicated staff was illustrated by the reconstitution of the ICARDA collections after the civil war in Syria. If such processes and backups had not been in place, there is no doubt that a considerable amount of unique diversity of wheat, barley, grain legumes and forages originating from the fertile crescent and beyond would have been lost to the world [34].

## Figures and Tables

**Table 1:** Crops and accession numbers of CGIAR genebanks in December 2020.

Centre	Crop(s)	Total Accessions in 2020 [3]
AfricaRice	Rice	21,815
Alliance-Bioversity International	Banana	1624
Alliance-International Center for Tropical Agriculture (CIAT)	Beans, cassava, tropical forages	64,635
International Maize and Wheat Improvement Center (CIMMYT)	Maize, wheat	147,842
International Potato Center (CIP)	Potato, sweetpotato, Andean roots and tubers	18,156
International Center for Agricultural Research in Dry Areas (ICARDA)	Dryland cereals, grain legumes, temperate forages	152,609
World Agroforestry (ICRAF)	Trees	14,919
International Crops Research Institute for the Semi-Arid Tropics (ICRISAT)	Sorghum, millets, grain legumes	129,034
International Institute of Tropical Agriculture (IITA)	Cowpea, maize, legumes, banana, cassava, yam	34,774
International Livestock Research Institute (ILRI)	Tropical forages	18,662
International Rice Research Institute (IRRI)	Rice	132,140
**Grand Total**		**736,210**

**Table 2:** Performance targets adopted by CGIAR genebanks in 2014.

Key Performance Indicators and Targets			
No.	Area	Indicator	Target
1	Germplasm availability	% of collection clean of pathogens of quarantine risk, viable, and in sufficient quantity to be immediately available for international distribution from medium-term storage (or local distribution for some tree species).	90% of accessions available
2	Safety duplication	For seed crops: % of collection in long-term storage at two locations and also in Svalbard Global Seed Vault (except for tree species). For clonal crops: % of collection in long-term storage or in vitro in slow growth conditions or in cryopreservation at two locations.	90% of seed accessions safety duplicated 50% of clonal accessions in cryopreservation (intermediate target), 90% of accessions duplicated in vitro
3	Data completeness and availability	Passport Data Completeness Index (PDCI) [29]: quantification of the completeness of the passport data based on the absence or presence of data points (range 0–10)	PDCI > 6
4	Quality management	Implementation of a Quality Management System (QMS)	Eight minimum elements of QMS in place (see QMS section below)

## Data Availability

All data and related information may be found at www.genebanks.org (last accessed 24 November 2021).
